# Interspecific Associations between *Cycloneda sanguinea* and Two Aphid Species (*Aphis gossypii* and *Hyadaphis foeniculi*) in Sole-Crop and Fennel-Cotton Intercropping Systems

**DOI:** 10.1371/journal.pone.0131449

**Published:** 2015-08-04

**Authors:** Francisco S. Fernandes, Francisco S. Ramalho, José B. Malaquias, Wesley A. C. Godoy, Bárbara Davis B. Santos

**Affiliations:** 1 Unidade de Controle Biológico, Embrapa Algodão, Campina Grande, Paraiba, Brazil; 2 Universidade de São Paulo (USP), Escola Superior de Agricultura “Luiz of Queiroz”, ESALQ, Departamento de Entomologia, Piracicaba, São Paulo, Brazil; Institute of Vegetables and Flowers, Chinese Academy of Agricultural Science, CHINA

## Abstract

Aphids cause significant damage to crop plants. Studies regarding predator-prey relationships in fennel (*Foeniculum vulgare* Mill.) and cotton (*Gossypium hirsutum* L.) crops are important for understanding essential ecological interactions in the context of intercropping and for establishing pest management programs for aphids. This study evaluated the association among *Hyadaphis foeniculi* (Passerini) (Hemiptera: Aphididae), *Aphis gossypii* Glover (Hemiptera: Aphididae) and *Cycloneda sanguinea* (L.) (Coleoptera: Coccinellidae) in cotton with coloured fibres, fennel and cotton intercropped with fennel. Association analysis was used to investigate whether the presence or absence of prey and predator species can indicate possible interactions between aphids and ladybugs. Significant associations among both apterous and alate *H*. *foeniculi* and *C*. *sanguinea* were observed in both the fennel and fennel-cotton intercropping systems. The similarity analysis showed that the presence of aphids and ladybugs in the same system is significantly dependent on the type of crop. A substantial amount of evidence indicates that the presence of the ladybug *C*. *sanguinea*, is associated with apterous or alate *A*. *gossypii* and *H*. *foeniculi* in fennel-cotton intercropping system. We recommend that future research vising integrated aphid management taking into account these associations for take decisions.

## Introduction

Ecological associations among insects can occur at various levels of organization, including within an organism, such as a plant, at the population level, or within communities [1]. Some insect species in the family Aphididae have a clear and primitive association with angiosperm plants and cause significant damage to them [2]. The aphid *Aphis gossypii* Glover (Hemiptera: Aphididae) begins to damage the leaves of cotton plants (*Gossypium hirsutum* L.) when they are in the vegetative phase. This behaviour may cause significant economic losses in cotton [3].

The aphid *Hyadaphis foeniculi* (Passerini) (Hemiptera: Aphididae) occurs on fennel plants (*Foeniculum vulgare* Mill.) when the ambient temperature increases and the plants begin to flower [4]. This behaviour generally results in economic losses and discourages the growth of fennel in northeast Brazil [4]. Fennel generally flowers at a different time than cotton. This can contribute to the permanence and association of predators in both crops; many of them feed on arthropod species that occur on the flowers and leaves of the plants. One of the main predators on cotton and fennel is the ladybug, *Cycloneda sanguinea* (L.) (Coleoptera: Coccinellidae). The abundance of this species depends on the abundance of aphids on both the leaves and fruits [5]. However, the study of these insects within intercropping system is incipient.

It is known that intercropping provides real conditions for the establishment of natural enemies in agricultural systems [5]. It has been recognized as valuable strategy to increase the density of predators, including ladybugs, to control aphid species [6] since the crop patterns may provide planting one species while harvesting the other one, taking into account partial life cycles overlapping. Co-occurrence between aphids and ladybugs has frequently been documented in different plant species, providing evidences of predator prey interaction [7, 8].

Studies regarding predator-prey relationships in fennel and cotton crops are important for understanding essential ecological interactions in the context of intercropping and for establishing pest management programs for aphids. This study evaluated the association among *H*. *foeniculi*, *A*. *gossypii* and *C*. *sanguinea* in fennel, cotton with coloured fibres and a fennel-cotton intercropping system. The information gleaned from this study will be useful in taking strategic pest control decisions.

## Materials and Methods

### Study location, cotton and fennel cultivars

This 3-yr study was carried out at an experimental farm of the Embrapa Algodão, Lagoa Seca, Paraiba, Brazil (latitude 7° 11’ 56”, longitude 35° 52’ 32”, elevation 635 m) during the 2009, 2010, and 2011 growing seasons. A naturally coloured cotton cultivar (BRS Safira) and a cultivar (Montadas) of fennel were planted on dark latossoil, under dryland conditions. The rainfall during the study were 109.2, 84.1, and 121.3 mm in 2009, 2010, and 2011 grown seasons, respectively. No specific permission was required for this location because it is a Research Station of the Embrapa Algodão. This field study did not involve endangered or protected species.

### Field plots and experimental design

Fennel and cotton plots in sole (rows of fennel alone or rows of cotton alone) or intercropping systems (two rows of fennel alternating with three rows of cotton) were installed following the same experimental design as the optimum arrangement proposed by Ramalho, et al. [4], i.e a randomized block design was used with three treatments: 1) two rows of fennel: three rows of cotton, 2) sole fennel, and 3) sole cotton, with four replications. The area of each experimental unit was 462 m^2^. In sole fennel, the rows were uniformly spaced in 1.50 m apart and in sole cotton, the rows were uniformly spaced in 1.00 m apart. Intercropping systems were designed with rows of fennel spaced in 1.50 m apart. The space between the fennel row and the adjacent cotton row was 1.50 m, and the cotton rows were spaced in 1.00 m apart. Field plots were planted in the first week of May, 2009, between the second and third weeks of May, 2010 and between the first and second weeks of June, 2011. Fennel plants were transplanted in 2009 and pruned during the 2010 and 2011 growing seasons. In the three years of the study, the cotton plants were not pruned, but were planted in sole-crop and intercropping systems. Each year, the cotton plants were removed after the last harvest.

### Arthropod species sampling

The presence of *H*. *foeniculi* and *A*. *gossypii*, both apterous and alate, and their natural enemy *C*. *sanguinea* were observed weekly in the sole fennel, sole cotton and intercropping systems, sampling 15 whole plants per plot beginning 18 d after plant emergence and continuing until the first open bolls appeared (125 d). In 2009, evaluations were performed between 55 and 191 days after the fennel was transplanted, or between 25 and 130 days after the cotton plants emerged. In 2010, the evaluations were performed between 34 and 188 days after the fennel was pruned, or between 22 and 147 days after the cotton plants emerged. In 2011, the evaluations were performed between 26 and 180 days after the fennel was pruned, or between 25 and 165 days after the cotton plants emerged. In 2009 and 2011 the plants were randomly marked before the evaluations. In 2010 the plants were not marked before the study but the evaluations were still randomly performed within the plots. The periods of study were larger in the fennel crop than cotton crop, because the fennel aphids usually attack the fennel in reproductive phase (flowers and seeds), while the cotton aphids usually attack the cotton in vegetative phase (leaves). In this sense, the study of the two aphid species present in intercropping systems over the time was important to consider the predator sustainability, taking into account the analyse of its presence in the same or different occurrences.

### Insect test of association

The interspecific associations of *A*. *gossypii* (apterous or alate) *versus C*. *sanguinea*, *H*. *foeniculi* (apterous or alate) *versus C*. *sanguinea* and *A*. *gossypii* (apterous or alate) *versus H*. *foeniculi* (apterous or alate) in both sole crops and intercropping systems were investigated using a presence-absence matrix (*M*_*2x2*_) for insects [9] that tracks weekly abundance estimates for three years. The time intervals when both species (*A* and *B*) occurred in a crop population (*a*), only species *A* occurred (*b*), only species *B* occurred (*c*) and neither species *A* nor *B* occurred (*d*) were recorded. *A* was considered to be the primary species (i.e., the most abundant), and *B* the secondary species. The sum (*N*) of all time intervals in which the species were present or absent was organized in a contingency table, equation: $M=\left|\begin{array}{cc} a & b\\ c & d \end{array}\right|$. More details on how analysis insect test of association using survey data you see in S1 Appendix.

### Jaccard dissimilarity and species cluster by ward method

The Jaccard [10] similarity index was computed from binary data regarding the presence or absence of insects (i.e., aphids and ladybugs) on plants from each data set. The values zero and one were attributed to the absence and presence, respectively, of the prey and natural enemy. This index provides a comparison between the temporal proximity of the occurrence of aphids and ladybugs. The Jaccard distance matrix for the clusters of insects was constructed via the Ward method. The presence–absence matrix (M) for insects and distance matrix you see in S2 Appendix, based on the Jaccard similarity or dissimilarity.

### Statistical analysis

The interspecific associations among insect species were quantified by Pearson’s χ^2^ using the software R (version 2.3.1). The dissimilarity and clustering of species were analysed using PROC distance of Sas [11] (method = djaccard and cluster: method = ward).

## Results

### Interspecific association of *C*. *sanguinea* with *H*. *foeniculi*

In sole fennel plots, both apterous and alate aphids co-occurred with ladybugs in 2009, 2010 and 2011 (Table 1). However, in 2009 and 2011, for the fennel-cotton intercropping system, significant associations were only observed among *H*. *foeniculi*, both apterous and alate, and *C*. *sanguinea* (Table 1).

**Table 1:** Association between *C*. *sanguinea* and *H*. *foeniculi* (apterous and alate) found in sole fennel and fennel-cotton intercropping systems.

Cropping system	Growing season	Relationship	Association			
			N	χ^2^	*P*	DF
Sole fennel	2009	Aphid (apterous) x Ladybug	21	17.23	0.0000	1
		Aphid (alate) x Ladybug	21	17.23	0.0000	1
	2010	Aphid (apterous) x Ladybug	23	10.40	0.0013	1
		Aphid (alate) x Ladybug	23	11.19	0.0008	1
	2011	Aphid (apterous) x Ladybug	23	10.14	0.0014	1
		Aphid (alate) x Ladybug	23	7.99	0.0047	1
Intercropping system	2009	Aphid (apterous) x Ladybug	21	10.10	0.0015	1
		Aphid (alate) x Ladybug	21	4.95	0.0261	1
	2010	Aphid (apterous) x Ladybug	23	2.27	0.1316	1
		Aphid (alate) x Ladybug	23	1.03	0.3106	1
	2011	Aphid (apterous) x Ladybug	23	9.51	0.0020	1
		Aphid (alate) x Ladybug	23	3.88	0.0487	1

*P <* 0.05 = The species co-occurred were associated in the same sample interval; *P >* 0.05 = The species occurred in a different time interval (i.e., they were independent).

### Interspecific association of *C*. *sanguinea* with *A*. *gossypii*

Our results indicated that in sole cotton plots the ladybug and *A*. *gossypii* co-occurred during 2010 and 2011 (Table 2). In the cotton-fennel intercropping system, similar results were found for *C*. *sanguinea* and *A*. *gossypii* in 2010 and 2011 but not for *C*. *sanguinea* and alate *A*. *gossypii* in 2010 (Table 2). In the both crop systems, we also found no co-occurrence between *C*. *sanguinea* and *A*. *gossypii* in 2009 (Table 2).

**Table 2:** Association between *C*. *sanguinea* and *A*. *gossypii* (apterous and alate) found in sole cotton and coloured cotton-fennel intercropping systems.

Cropping system	Growing season	Relationship	Association			
			N	χ^2^	*P*	DF
Sole cotton	2009	Aphid (apterous) x Ladybug	16	1.78	0.1824	1
		Aphid (alate) x Ladybug	16	0.02	0.8892	1
	2010	Aphid (apterous) x Ladybug	21	8.69	0.0032	1
		Aphid (alate) x Ladybug	21	5.70	0.0170	1
	2011	Aphid (apterous) x Ladybug	19	13.65	0.0002	1
		Aphid (alate) x Ladybug	19	3.88	0.0487	1
Intercropping system	2009	Aphid (apterous) x Ladybug	16	2.35	0.1256	1
		Aphid (alate) x Ladybug	16	0.87	0.3502	1
	2010	Aphid (apterous) x Ladybug	21	10.98	0.0009	1
		Aphid (alate) x Ladybug	21	2.17	0.1407	1
	2011	Aphid (apterous) x Ladybug	19	11.20	0.0008	1
		Aphid (alate) x Ladybug	19	4.86	0.0274	1

*P <* 0.05 = The species were associated co-occurred in the same sample interval; *P >* 0.05 = The species occurred in different time intervals (i.e., they were independent).

### Relationship of *H*. *foeniculi* and *A*. *gossypii* in an intercropping system

The apterous and alate aphids *H*. *foeniculi* and *A*. *gossypii* co-occurred in 2009 (Table 3). However, there was no association between apterous *A*. *gossypii* and apterous *H*. *foeniculi* or between alate *A*. *gossypii* and alate *H*. *foeniculi* in 2010 or 2011 in the intercropping system (Table 3).

**Table 3:** Independence of *H*. *foeniculi* (apterous or alate) and *A*. *gossypii* (apterous or alate) in the intercropping systems.

Species	Intercropping system				Growing season
	Association				
	N	χ^2^	*P*	DF	
Cotton aphid x fennel aphid (apterous)	25	11.06	0.0009	1	2009
Cotton aphid x fennel aphid (alate)	25	5.94	0.0148	1	2009
Cotton aphid x fennel aphid (apterous)	28	0.02	0.8848	1	2010
Cotton aphid x fennel aphid (alate)	28	0.45	0.5027	1	2010
Cotton aphid x fennel aphid (apterous)	24	1.43	0.2311	1	2011
Cotton aphid x fennel aphid (alate)	24	0.80	0.3711	1	2011

*P <* 0.05 = The species were associated co-occurred in the same sample interval; *P >* 0.05 = The species occurred in different time intervals (i.e., they were independent).

### Jaccard dissimilarity of apterous and alate *H*. *foeniculi* and *C*. *sanguinea* in the sole fennel and fennel-cotton intercropping systems

According to the Jaccard matrix, the time interval in which alate *H*. *foeniculi* was found in fennel intercropped with cotton was similar to the time interval in which *C*. *sanguinea* was found in sole fennel (0.33333).

**Fig 1:**
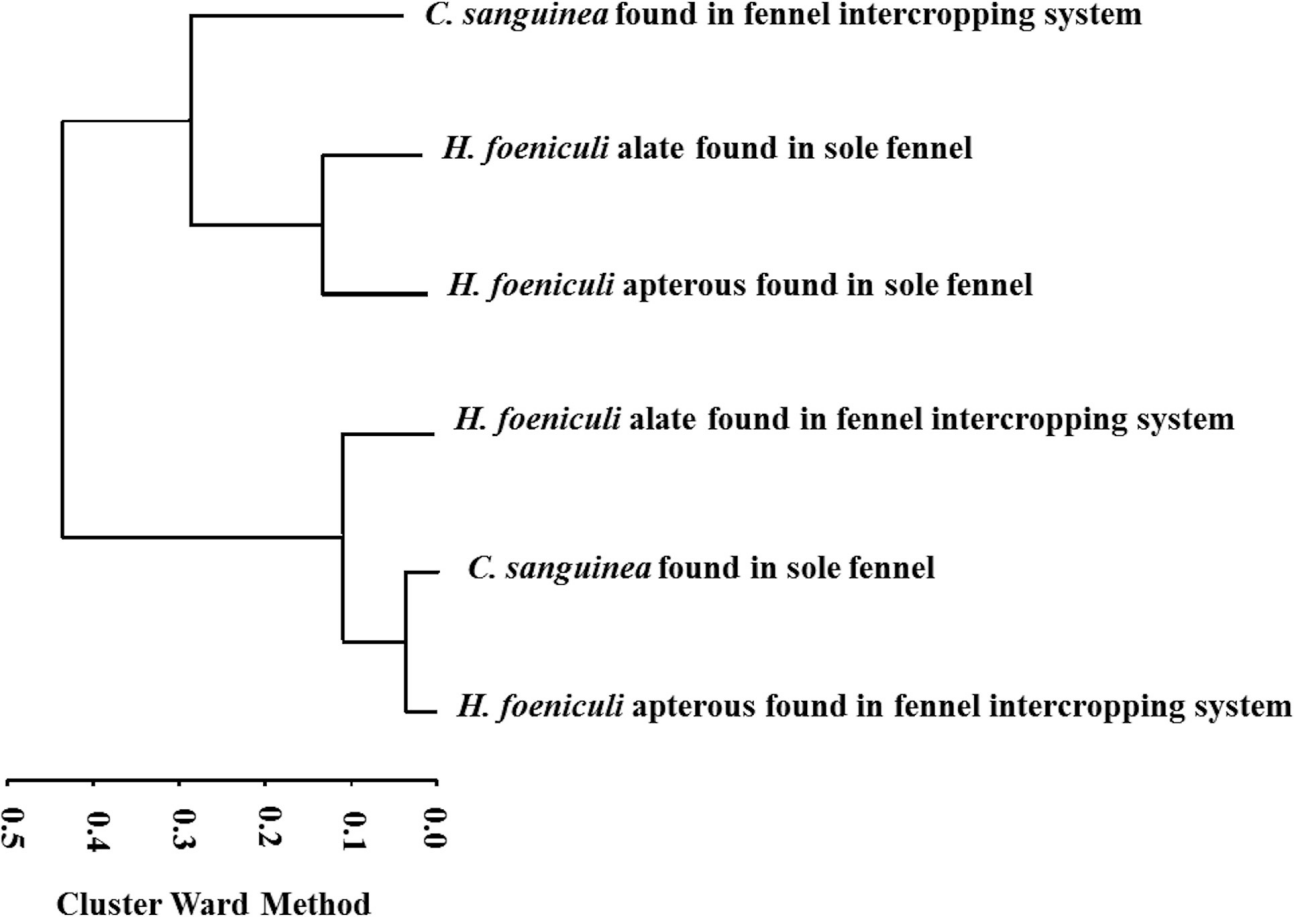
Co-occurrence of *H*. *foeniculi* and *C*. *sanguinea* in sole fennel and intercropped fennel-cotton.

Observing the dendrogram from right to left and inserting a cut close to 0.3, we identify two well-defined groups each with two subgroups via the cluster by Ward quadratic sums (Fig 1). These sums indicate how frequently insects co-occurred through time and across cropping systems.

Comparing the subgroups within the dendrogram from bottom to top, in the first subgroup we observe a cluster of apterous and alate *H*. *foeniculi* from the fennel-cotton intercropping system and *C*. *sanguinea* from the sole fennel system. The second subgroup contains a cluster of apterous and alate *H*. *foeniculi* from the sole fennel system and *C*. *sanguinea* from the fennel-cotton intercropping system (Fig 1). These results indicate that, within each subgroup, the insects co-occurred regardless of the year.

### Jaccard dissimilarity for apterous and alate *A*. *gossypii* and *C*. *sanguinea* in sole cotton and fennel-cotton intercropping systems

According to the Jaccard matrix, the time interval in which apterous *A*. *gossypii* was found in cotton intercropped with fennel was the same as that in which alate *A*. *gossypii* was present in sole cotton (0.00000). The greatest distance for insect occurrence was 1.00000.

**Fig 2:**
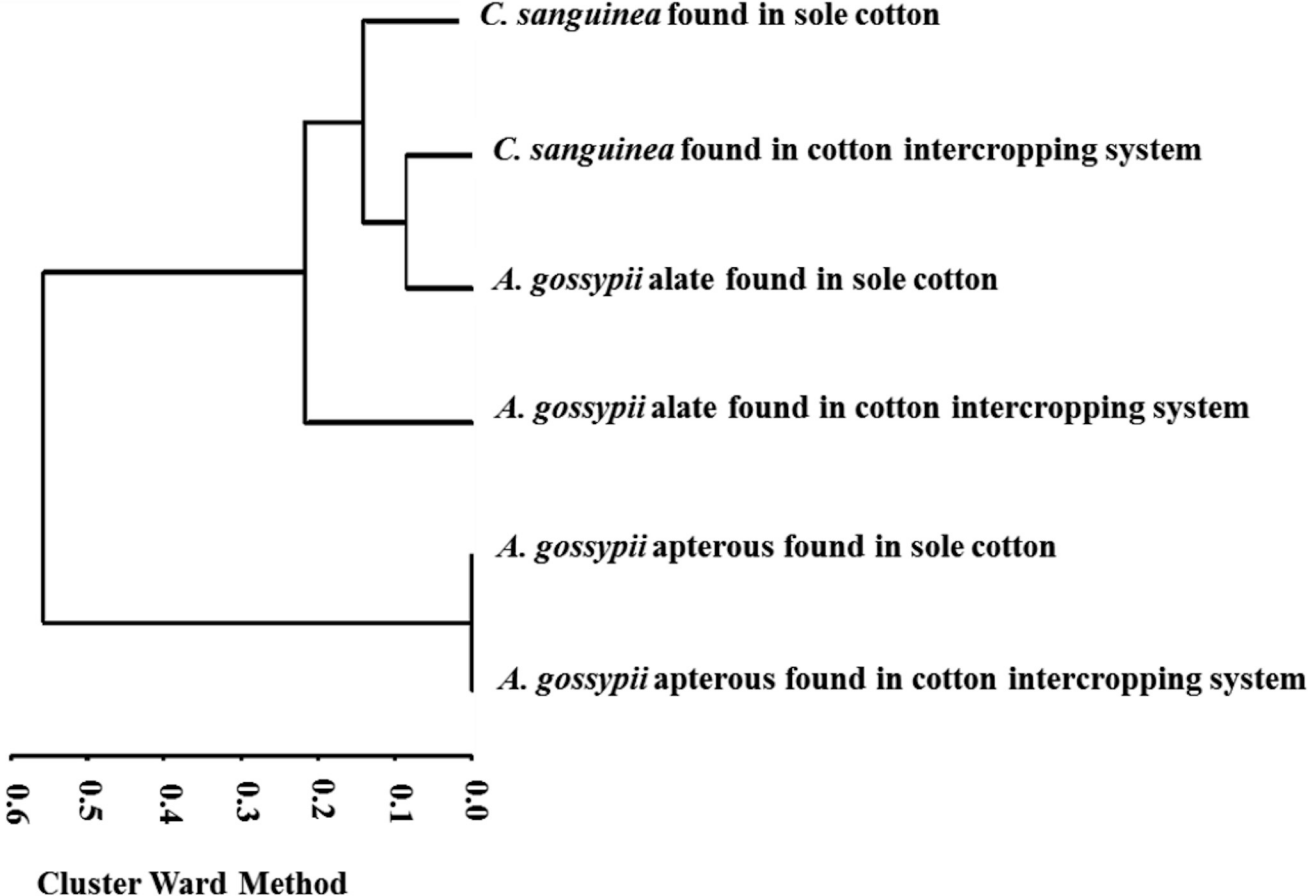
Co-occurrence of *A*. *gossypii* and *C*. *sanguinea* studied in sole cotton and intercropped fennel-cotton.

Observing the dendrogram from right to left and inserting a cut near 0.28, we see two well-defined groups containing one (*A*. *gossypii* apterous from sole cotton + *A*. *gossypii* apterous from cotton intercropping system) and three subgroups within the cluster by Ward quadratic sums (Fig 2). These sums indicate how many times apterous and alate *A*. *gossypii* spatially co-occurred with *C*. *sanguinea*, whether at the same time or not, in the sole cotton and fennel-cotton intercropping systems.

Comparing the dendrogram from top to bottom shows that in the first subgroup there are clusters of *C*. *sanguinea* found in sole cotton, *C*. *sanguinea* found in the cotton-fennel intercropping system, alate *A*. *gossypii* found in sole cotton and alate *A*. *gossypii* found in intercropping system (Fig 2). In the second subgroup there are clusters of apterous *A*. *gossypii* found in sole cotton and apterous *A*. *gossypii* found in cotton intercropped with fennel. These results demonstrated that insects within a subgroup co-occurred independently of the year. Based on the insects’ co-occurrence, we suggest the following hypotheses: the predator found in sole cotton and the predator found in the cotton rows of the intercropping system may migrate between crops in response to aphids present on these crops at the same time (Fig 2).

## Discussion

The results regarding the co-occurrence of *C*. *sanguinea* with *A*. *gossypii* and *H*. *foeniculi* in the intercropping system demonstrated that the occurrence of the predatory ladybug was dependent on the presence of both prey species (i.e., *A*. *gossypii* and *H*. *foeniculi*) within the cropping system. When both prey were present, the predator remained associated with *H*. *foeniculi*, whether apterous or alate. This was most likely due to the higher population density of *H*. *foeniculi* than *A*. *gossypii*, as previously reported by Fernandes, et al. [12,13]. Number of cotton aphids found on a cotton plant for the entire cotton season (11,444 aphids) was significantly higher than the number found on the intercropped plots (8,106 aphids) [13]. The number of apterous aphids and alate aphids found per fennel plant in the sole fennel (27,922 apterous and 703 alate aphids) for the entire season were significantly higher than the number found in the fennel-cotton intercropped system (13,012 apterous and 369 alate aphids) [12]. The highest density of ladybugs associated with the suppression of cotton and fennel aphids populations was found in the intercropping system consisting of three rows of cotton with two rows of fennel (5 ladybugs/cotton plant and 20 ladybugs/fennel plant) [3,4]. These associations were also found in a study by Fritz, et al. [14]. They obtained similar results regarding the time of occurrence of insects, and suggested that variation in plants allow stronger positive associations between insect species.

Aphids are usually positively associated with host plants that have extra floral nectaries [15,16]. However, the results found for fennel aphids *versus* cotton aphids in the 2010 and 2011 growing seasons indicated that they not co occurred within intercropping system. This may have happened due the distinct time of flowering between two plants species. Similarly results for aphids study within crops were found by Lamb [17] that observed different time of occurrence of *A*. *gossypii*, *Aphis craccivora* Koch (Hemiptera: Aphididae), *Tetraneura nigriabdominalis* Sazaki (Hemiptera; Aphididae) and other aphids species less frequently. The possibly explanation used in his paper was that many aphids can migrate from different location to another by influence of wind and they can choose the best host plant maybe in different location and time. However more information to explain it is needed for best understand of aphid occurrence in fennel and cotton intercropping systems.

Our results indicated that temporal occurrence of aphid species *A*. *gossypii* and *H*. *foeniculi* is potentially favourable for the conservation, reproduction, feeding and maintenance of the predator, *C*. *sanguinea*, in the fennel-cotton intercropping system. The increase in synchrony of immature and adult stages of ladybugs with the prey *A*. *gossypii* and *H*. *foeniculi* prevents local extinction of the predator, increasing the potential for biological control of both pests in the cotton-fennel intercropping system [18]. Furthermore, based on the time series of abundances, the ladybug and cotton and fennel aphids showed similar population fluctuations and may have a close association with the plant canopy [19].

Our results regarding changes in predator abundance in response to *H*. *foeniculi* apterous and alate aphids in the fennel-cotton intercropping system in the 2010 growing season (Table 1), as well as the temporal abundance of *H*. *foeniculi* and *C*. *sanguinea* in the 2011 growing season, are essential for the establishment of future intercropping systems. They suggest the following hypothesis: the cotton-fennel intercropping system can contribute to the maintenance of the ladybug population, thereby reducing the aphid (i.e., *A*. *gossypii* and *H*. *foeniculi*) populations. Studies conducted by Ramalho, et al. [3,4] demonstrated that cotton-fennel intercropping systems support larger numbers of *C*. *sanguinea* and fewer aphids than sole systems. Similarly, a study conducted by Ma, et al. [20] found greater aphid abundances in a sole plot than in an intercropping plot. The intercropping system can result in less damage to plants and higher productivity for the farmer [21].

The Jaccard index can also be used to quantify the similarity of different habitats [22]. The abundance and relative proportions of the functional foods of insect groups may vary with the habitat [23]. Observations the similarity between the number of occurrences of *C*. *sanguinea* in the sole crop and apterous aphids in the fennel-cotton intercropping system (0.33333), as well as the similarity between the number of occurrences of apterous aphids in rows of the fennel-cotton intercropping systems and the number of occurrences of alate aphids in the same crop system (0.33333) shows that throughout the year there was similarity between associations of these insects. A similar conclusion can be reached based on the proximity of the number of occurrences of apterous *A*. *gossypii* in rows of the fennel-cotton intercropping system and the number of occurrences of apterous *A*. *gossypii* in the sole crop. Like many natural enemies, adult ladybugs are transient predators, foraging within several habitats during the growing season [24]. For this reason, their diversity and abundance are expected to depend on both the abundance of prey within crop habitat and the structure and composition of the surrounding landscape [25]. However, studies reported by Altiere [26] within-field or field-edge, showed that the presence of aphids and ladybugs in the same system is a function of the crop. Therefore, the similarity analysis showed that the presence of aphids and ladybugs in the same system is significantly dependent on the type of crop.

Although Taylor [27] and Hodek and Michaud [28] have suggested that the chance of an encounter between aphids and ladybird beetles depends on several factors, especially spatial distribution and wind, our cluster analyses (Figs 1 and 2) demonstrated subgroups containing apterous and alate aphids and their natural enemy, *C*. *sanguinea*, within the crop system. This supports the hypothesis that the fennel-cotton intercropping system allows the immigration of beneficial insects. The migration of *C*. *sanguinea* most likely occurred in response to the presence of *H*. *foeniculi* or *A*. *gossypii*, because it was observed in differing time periods and intercropping systems. According to Grez, et al. [29], in agricultural crop systems, many species of ladybugs are not affected by the composition of the border crop. However, the aphid population may be affected by physical barriers from crops and natural enemies in a fennel-cotton intercropping system [3, 4]. Another factor that affects aphids and their natural enemies is the difference in nutritional composition of each crop or habitat [30]. According to Osawa [31], ladybug populations show a pattern of dispersal between subpopulations and the establishment of populations is determined by evaluation and accessibility of aphid colonies. This is important in our context because, if farmers maintain different crops in which beneficial insects are present, reduction of aphids is highly probable and will likely result in a significant increase in productivity [13].

To conclude, there is substantial evidence to indicate that the presence of the *C*. *sanguinea* is associated with apterous or alate *A*. *gossypii* and *H*. *foeniculi* in fennel-cotton intercropping system. We recommend that future research vising integrated aphid management taking into account these associations for take decisions.

## Supporting Information

S1 Appendix Appendix 1. (DOCX)

S2 Appendix Appendix 2. (DOCX)
