# Supplementary material for: Interspecific Associations between Cycloneda sanguinea and Two Aphid Species (Aphis gossypii and Hyadaphis foeniculi) in Sole-Crop and Fennel-Cotton Intercropping Systems
Source: PLoS One. 2015 Aug 4;10(8):e0131449. doi: 10.1371/journal.pone.0131449 (PMC4524726; doi:10.1371/journal.pone.0131449)

**Appendix 1.**  How to analyse insect test of association using survey data

For this case, we compare the presence (1) or absence (0) of insect’s pair (aphid and natural enemy) apart of crop system, crop season, aphid stage or insects density (i.e. data set in supplementary Tables 1 and 2).

Supplementary Table 1. Presence (1) or absence of apterous cotton aphid and ladybird (*C. sanguinea*) in sole cotton in 2009 season

| Plant age | *A. gossypii* (apterous) | *C. sanguinea* |
| --- | --- | --- |
| 25 | 0 | 0 |
| 32 | 1 | 0 |
| 39 | 1 | 1 |
| 46 | 1 | 1 |
| 53 | 1 | 1 |
| 60 | 1 | 1 |
| 67 | 1 | 1 |
| 74 | 1 | 1 |
| 81 | 1 | 1 |
| 88 | 1 | 1 |
| 95 | 1 | 1 |
| 102 | 1 | 1 |
| 109 | 1 | 0 |
| 116 | 1 | 0 |
| 123 | 1 | 0 |
| 130 | 1 | 0 |

According to each data set, we following the step:

Ludwig and Reynolds (1988) (Contingence table) – Two species case:

|  |  | Aphid |  |  |
| --- | --- | --- | --- | --- |
| *Cycloneda* |  | Presence | absence |  |
|  | Presence | a: | b: | sum |
|  | absence | c: | d: | sum |
|  |  | sum | sum | N |

The analysis can be run with the equations below:

Step 1:

|  |  | Aphid |  |  |
| --- | --- | --- | --- | --- |
| *Cycloneda* |  | Presence | absence |  |
|  | Presence | 10 | 5 | 15 |
|  | absence | 0 | 1 | 1 |
|  |  | 10 | 6 | 16 |

Step 2: Question – Are apterous aphids and *C. sanguinea* occurring in sole crop within 2009 season independently?

Step 3: Compute test statistic:


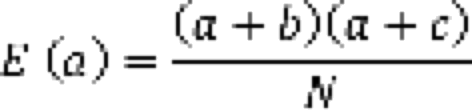


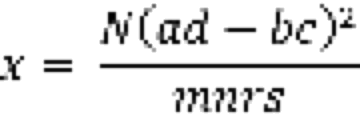


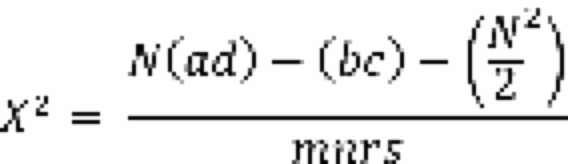


Supplementary Table 2. Presence (1) or absence of alate cotton aphid and ladybird (*C. sanguinea*) in sole cotton in 2009 season

| Plant age | *A. gossypii* (alate) | *C. sanguinea* |
| --- | --- | --- |
| 25 | 0 | 0 |
| 32 | 1 | 0 |
| 39 | 0 | 1 |
| 46 | 0 | 1 |
| 53 | 1 | 1 |
| 60 | 0 | 1 |
| 67 | 1 | 1 |
| 74 | 1 | 1 |
| 81 | 1 | 1 |
| 88 | 1 | 1 |
| 95 | 1 | 1 |
| 102 | 1 | 1 |
| 109 | 0 | 0 |
| 116 | 1 | 0 |
| 123 | 1 | 0 |
| 130 | 1 | 0 |

According to each data set, we following the step:

Step 1:

|  |  | Aphid |  |  |
| --- | --- | --- | --- | --- |
| *Cycloneda* |  | Presence | absence |  |
|  | Presence | 7 | 4 | 11 |
|  | absence | 3 | 2 | 5 |
|  |  | 10 | 6 | 16 |

Step 2: Question – Are apterous aphid and *C. sanguinea* occurring in sole crop within 2009 season independently?

Step 3: Compute test statistic


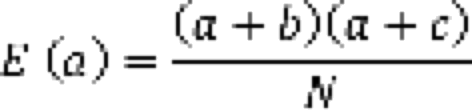


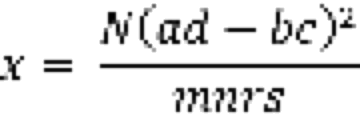


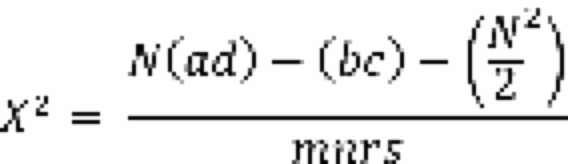


This procedure was also made to other crop system in other years.

The analysis can be run with the equations below:

Step 1:

|  |  | Aphid |  |  |
| --- | --- | --- | --- | --- |
| *Cycloneda* |  | Presence | absence |  |
|  | Presence | a: | b: | m |
|  | absence | c: | d: | n |
|  |  | r | s | N |

Step 2: Question – Are apterous aphid and *C. sanguinea* occurring in sole crop within 2009 season independently?

Step 3: Compute test statistic


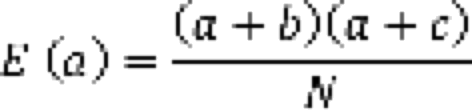


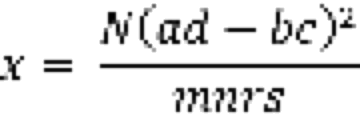


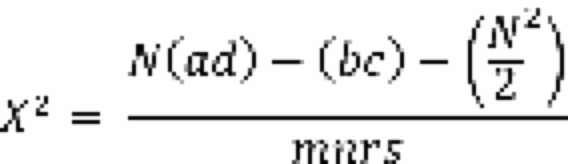

Supplement: S1 Appendix — (DOCX) [file pone.0131449.s001.docx]
