# Supplementary material for: Interspecific Associations between Cycloneda sanguinea and Two Aphid Species (Aphis gossypii and Hyadaphis foeniculi) in Sole-Crop and Fennel-Cotton Intercropping Systems
Source: PLoS One. 2015 Aug 4;10(8):e0131449. doi: 10.1371/journal.pone.0131449 (PMC4524726; doi:10.1371/journal.pone.0131449)
Supplement: S2 Appendix — (DOCX) [file pone.0131449.s002.docx]

**Appendix 2.** The presence-absence matrix (M) for insects and distance matrix in appendix, based on the Jaccard similarity or dissimilarity

**Jaccard dissimilarity and cluster by ward method**

**1) Fennel**

Occurrence of insects within sole fennel and fennel-cotton intercropping systems

| Insect specie | Crop system | Crop season | | |
| --- | --- | --- | --- | --- |
|  |  | 2009 | 2010 | 2011 |
| Apterous *H. foeniculi* | Intercropping | 8 | 19 | 12 |
|  | Sole | 9 | 21 | 13 |
| Alate *H. foeniculi* | Intercropping | 8 | 21 | 12 |
|  | Sole | 9 | 19 | 14 |
| *C. sanguinea* | Intercropping | 7 | 18 | 9 |
|  | Sole | 8 | 19 | 11 |

Input in Sas software:

/* Hyadaphis plant age occurrance in 2009-2011- distance Jaccard*/

data Distance fennel;

input Specie $ year 2009 year 2010 year 2011;

cards;

Hyadahisfoeniculiapc 8 19 12

Hyadahisfoeniculiaps 9 21 13

Hyadahisfoeniculialc 8 21 12

Hyadahisfoeniculials 9 19 14

Cyclonesanguineac 7 18 9

Cyclonesanguineas 8 19 11

;

proc print;

run;

proc distance data=Distance fennel method=djaccard out=Exit;

var anominal (Year2009 year2010 year2011);

run;

proc print data=Exit;

run;

Jaccard matrix distance for *H. foeniculi* and its predator, (*C. sanguinea*) in sole fennel and fennel- cotton intercropping systems (2009, 2010 and 2011 growing seasons).

| Species | Aap (Int) | Aap (Sol) | Aal (Int) | Aal (Sol) | Spot (Int) | Spot (Sol) |
| --- | --- | --- | --- | --- | --- | --- |
| Aap (Int) | 0.00000 | 1.00000 | 0.33333 | 0.66667 | 1.00000 | 0.33333 |
| Aap (Sol) | 1.00000 | 0.00000 | 0.66667 | 0.66667 | 1.00000 | 1.00000 |
| Aal (Int) | 0.33333 | 0.66667 | 0.00000 | 1.00000 | 1.00000 | 0.66667 |
| Aal (Sol) | 0.66667 | 0.66667 | 1.00000 | 0.00000 | 1.00000 | 0.66667 |
| Spot (Int) | 1.00000 | 1.00000 | 1.00000 | 1.00000 | 0.00000 | 1.00000 |
| Spot (Sol) | 0.33333 | 1.00000 | 0.66667 | 0.66667 | 1.00000 | 0.00000 |

Aap (Int) = *H. foeniculi* (apterous) found in fennel-cotton intercropping system; Aap (Sol) = *H. foeniculi* (apterous) found in sole fennel; Aal (Int) = *H. foeniculi* (alate) found in fennel-cotton intercropping system; Aal (Sol) = *H. foeniculi* (alate) found in sole fennel; Spot (Int) = *C. sanguinea* found in fennel-cotton intercropping system; Spot s (Sol) = *C. sanguinea* found in sole fennel.

Input in Sas software:

/*Compareaphid Hyadaphis plant age occurrance in 2009-2011-Getting Cluster with Jaccard distance*/

data DistanceFennel;

input Insect $ Hyadahisfoeniculiapc Hyadahisfoeniculiaps Hyadahisfoeniculialc Hyadahisfoeniculials Cyclonesanguineac Cyclonesanguineas;

cards;

HFapc 0.00000 1.00000 0.33333 0.66667 1.00000 0.33333

HFaps 1.00000 0.00000 0.66667 0.66667 1.00000 1.00000

HFalc 0.33333 0.66667 0.00000 1.00000 1.00000 0.66667

HFals 0.66667 0.66667 1.00000 0.00000 1.00000 0.66667

Cyclc 1.00000 1.00000 1.00000 1.00000 0.00000 1.00000

Cycls 0.33333 1.00000 0.66667 0.66667 1.00000 0.00000

;

proc cluster method=complete data=DistanceFennel outtree=tree2;

VAR Hyadahisfoeniculiapc Hyadahisfoeniculiaps Hyadahisfoeniculialc Hyadahisfoeniculials Cyclonesanguineac Cyclonesanguineas;

ID Insect;

RUN;

PROC TREE DATA=tree2 SPACES=2;

ID Insect;

RUN;

proc cluster method=ward data=Distancefennel outtree=tree2;

VAR Hyadahisfoeniculiapc Hyadahisfoeniculiaps Hyadahisfoeniculialc Hyadahisfoeniculials Cyclonesanguineac Cyclonesanguineas;

ID Insect;

RUN;

PROC TREE DATA=tree2 SPACES=2;

ID Insect;

RUN;

**2) Cotton**

Occurrence of insects within sole cotton and cotton- fennel intercropping systems

| Insect specie | Crop system | Crop season | | |
| --- | --- | --- | --- | --- |
|  |  | 2009 | 2010 | 2011 |
| Apterous *A. gossypii* | Intercropping | 15 | 15 | 15 |
|  | Sole | 15 | 15 | 15 |
| Alate *A. gossypii* | Intercropping | 15 | 15 | 8 |
|  | Sole | 11 | 12 | 11 |
| *C. sanguinea* | Intercropping | 11 | 13 | 12 |
|  | Sole | 10 | 12 | 13 |

Input in Sas software

/*compare Aphis gossypii plant age occurrence in year 2009-2011-Getting Jaccard distance*/

**data** cotton;

input Specie $ year2009 year2010 year2011;

cards;

Aphisgossypiiapc 15 15 15

Aphisgossypiiaps 15 15 15

Aphisgossypiialc 12 11 8

Aphisgossypiials 11 12 11

Cyclonesanguineac 11 13 12

Cyclonesanguineas 10 12 13

;

proc print;

run;

proc distance data=cotton method=djaccard out=exit4;

var anominal (Ayear2009 year2010 year2011);

run;

proc print data=exit4;

run;

Jaccard matrix distance for *A. gossypii* and its predator, (*C. sanguinea*) in sole cotton and fennel-cotton intercropping systems (2009, 2010 and 2011 growing seasons)

| Species | Aap (Int) | Aap (Sol) | Aal (Int) | Aal (Sol) | Spot (Int) | Spot (Sol) |
| --- | --- | --- | --- | --- | --- | --- |
| Aap (Int) | 0.00000 | 0.00000 | 1.00000 | 1.00000 | 1.00000 | 1.00000 |
| Aap (Sol) | 0.00000 | 0.00000 | 1.00000 | 1.00000 | 1.00000 | 1.00000 |
| Aal (Int) | 1.00000 | 1.00000 | 0.00000 | 1.00000 | 1.00000 | 1.00000 |
| Aal (Sol) | 1.00000 | 1.00000 | 1.00000 | 0.00000 | 0.66667 | 0.66667 |
| Spot (Int) | 1.00000 | 1.00000 | 1.00000 | 0.66667 | 0.00000 | 1.00000 |
| Spot (Sol) | 1.00000 | 1.00000 | 1.00000 | 0.66667 | 1.00000 | 0.00000 |

Aap (Int) = *A. gossypii* (apterous) found in intercropped fennel-cotton; Aap (Sol) = *A. gossypii* (apterous) found in sole cotton; Aal (Int) = *A. gossypii* (alate) found in intercropped fennel-cotton; Aal (Sol) = *A. gossypii* (alate) found in sole cotton; Spot (Int) = *C. sanguinea* found in cotton-fennel intercropping system; Spot (Sol) = *C. sanguinea* found in sole cotton.

Input in Sas software

/*compare Aphis gossypii - occurrence in year 2009-2011-Getting Cluster with Jaccard distance*/

data cotton;

input Insect $ Aphisgossypiiapc Aphisgossypiiaps Aphisgossypiialc Aphisgossypiials Cyclonesanguineac Cyclonesanguineas;

cards;

AGpc 0.00000 0.00000 1.00000 1.00000 1.00000 1.00000

AGaps 0.00000 0.00000 1.00000 1.00000 1.00000 1.00000

AGalc 1.00000 1.00000 0.00000 1.00000 1.00000 1.00000

AGals 1.00000 1.00000 1.00000 0.00000 0.66667 0.66667

Cyclc 1.00000 1.00000 1.00000 0.66667 0.00000 1.00000

Cycls 1.00000 1.00000 1.00000 0.66667 1.00000 0.00000

;

proc cluster method=complete data=cotton outtree=tree2;

VAR Aphisgossypiiapc Aphisgossypiiaps Aphisgossypiialc Aphisgossypiials Cyclonesanguineac Cyclonesanguineas;

ID Insect;

RUN;

PROC TREE DATA=tree2 SPACES=2;

ID Insect;

RUN;

proc cluster method=ward data=Algodao outtree=tree2;

VAR Aphisgossypiiapc Aphisgossypiiaps Aphisgossypiialc Aphisgossypiials Cyclonesanguineac Cyclonesanguineas;

ID Insect;

RUN;

PROC TREE DATA=tree2 SPACES=2;

ID Insect;

RUN;
